# Bioactive Anti-Inflammatory Compounds and Therapeutic Strategies for Promoting Resolution

**DOI:** 10.3390/pharmaceutics18060687

**Published:** 2026-05-30

**Authors:** Dipa K. Israni, Mansi Shah, Heena Chauhan, Mumuxa Rathod, Bhupendra G. Prajapati, Supachoke Mangmool, Sudarshan Singh, Chuda Chittasupho

**Affiliations:** 1 Department of Pharmacology, LJ Institute of Pharmacy, LJ University, Ahmedabad 382210, Gujarat, India; dr.dipa.israni@ljku.edu.in (D.K.I.); mansi.shah_ljip@ljku.edu.in (M.S.); heena.chauhan@ljku.edu.in (H.C.); mumuxa.rathod@ljku.edu.in (M.R.); 2 Department of Pharmaceutics, Faculty of Pharmacy, Parul Institute of Pharmacy, Parul University, Waghodia, Vadodara 391760, Gujarat, India; bhupen27@gmail.com; 3 Faculty of Pharmacy, Chiang Mai University, Chiang Mai 50200, Thailand; supachoke.man@cmu.ac.th; 4 Office of Research Administration, Chiang Mai University, Chiang Mai 50200, Thailand

**Keywords:** bioactive compounds, inflammation resolution, oxidative stress, nanoformulation, pro-resolving mediators, personalized medicine

## Abstract

Inflammation plays a crucial role in defending the body against harmful stimuli and maintaining physiological balance; however, when it becomes chronic, it contributes to the pathogenesis of several long-term diseases, including autoimmune conditions, cardiovascular and neurodegenerative disorders, and various cancers. Although conventional anti-inflammatory drugs provide symptomatic relief, their long-term use is often associated with adverse side effects. This limitation has shifted scientific attention toward naturally occurring bioactive molecules with potent, safer anti-inflammatory activity. Dietary incorporation of phytopharmaceuticals, such as flavonoids, polyphenols, alkaloids, terpenoids, and fatty acids, has been shown to regulate immune and oxidative mechanisms and to modulate key inflammatory signaling cascades, including the NF-κB, mitogen-activated protein kinase (MAPK), and JAK/STAT pathways. These agents also influence cytokine secretion, NLRP3 inflammasome activation, and antioxidant defense mechanisms involving the Nrf2/HO-1 axis. The current review emphasizes the relevance of major natural plant products in therapy, like quercetin and rutin, resveratrol, glycyrrhizin, lycopene, and indole-3-carbinol. Moreover, recent progress in anti-inflammatory research has focused on novel resolution-based strategies that extend beyond inflammation and oxidative stress suppression. In addition, the review discusses innovations including nanoformulation-assisted targeted delivery, specialized pro-resolving lipid mediators such as resolvins and protectins, and microbiota-oriented therapeutic approaches. Additionally, the review highlights the integration of personalized medicine supported by multi-omics technologies to enhance treatment precision and clinical outcomes. By synthesizing findings from preclinical studies and clinical investigations, this work emphasizes the synergistic therapeutic potential of bioactive compounds from natural sources and resolution-enhancing techniques in restoring immune homeostasis and effectively mitigating chronic inflammation.

## 1. Introduction

Inflammation is a fundamental component of the innate immune response, acting as a defense mechanism against pathogens, tissue injury, and other harmful stimuli. Acute inflammation serves a protective role by eliminating invaders, activating immune cells, and initiating tissue repair. However, when inflammation becomes dysregulated or persists chronically, it contributes to the pathogenesis of numerous diseases, underscoring its dual nature: “protective in acute phases but deleterious when chronic” [1]. Many diseases are caused by chronic inflammation, such as rheumatoid arthritis (RA), a systemic autoimmune disorder that affects over 17.6 million individuals globally. About 2.3 million Americans suffer from chronic intestinal inflammation, such as inflammatory bowel disease (IBD), which includes Crohn’s disease and ulcerative colitis. Type 2 diabetes mellitus is characterized by low-grade systemic inflammation and affects over 537 million adults globally. Osteoarthritis, particularly involving the knee joint, affects an estimated 250 million people. Neuroinflammation is increasingly implicated in Alzheimer’s disease and other neurodegenerative conditions, contributing to over 55 million global dementia cases. Furthermore, inflammation-related mechanisms contribute to approximately 2.8 million new cancer cases annually, underscoring their role in tumorigenesis [2].

In cardiovascular disease, particularly atherosclerosis, inflammation initially acts protectively by removing oxidized low-density lipoproteins and repairing endothelial injury. However, chronic inflammation drives disease progression through foam cell formation, cytokine release, and plaque instability. Important biomarkers that are strongly linked to elevated cardiovascular risk include high-sensitivity C-reactive protein (hsCRP) and interleukin-6 (IL-6) [3]. In IBD, the inflammatory response maintains mucosal integrity and defends against microbial invasion through cytokines such as tumor necrosis factor-alpha (TNF-α), interleukin-1 beta (IL-1β), and IL-6 [4]. However, sustained activation leads to mucosal damage, ulceration, and epithelial dysfunction, resulting in chronic disease progression [5].

Chronic neurodegenerative illnesses, including Alzheimer’s disease and Parkinson’s disease, share common pathophysiological mechanisms. Oxidative stress and neuronal damage are caused by reactive oxygen and nitrogen species generated during inflammation. In multiple sclerosis (MS), autoimmune attacks on oligodendrocytes lead to demyelination, axonal degeneration, and glial scar formation, manifesting as neurological impairments [6,7]. A critical barrier to resolving chronic inflammation is the failure of endogenous pro-resolving pathways. Lipoxins, resolvins, protectins, and maresins are examples of specialized pro-resolving mediators (SPMs) that actively reduce inflammation by reducing neutrophil infiltration, enhancing macrophage-mediated efferocytosis, and promoting tissue repair. Dysregulated SPM signaling allows persistent cytokine production and leukocyte recruitment, contributing to diseases such as RA, IBD, atherosclerosis, and neurodegeneration [8].

Conventional anti-inflammatory agents, such as nonsteroidal anti-inflammatory drugs (NSAIDs) and corticosteroids, primarily inhibit pro-inflammatory pathways by inhibiting cyclooxygenase activity and cytokine expression. While effective in reducing the acute inflammatory response, these agents do not actively engage endogenous resolution pathways and may, in certain contexts, prolong inflammation by inhibiting lipid mediator class switching and impairing efferocytosis. In contrast, resolvins, SPMs derived from omega-3 fatty acids, promote resolution by engaging G protein-coupled receptors (GPCRs) (e.g., GPR32, ChemR23, GPR18) on immune cells. They reduce neutrophil influx, enhance efferocytosis, suppress cytokines, and support tissue regeneration [9,10]. Resolvins, such as resolvin D1 (RvD1), resolvin D2 (RvD2), and resolvin E1 (RvE1), have shown promise in preclinical and clinical models of chronic inflammation, including RA, diabetes, atherosclerosis, and neuroinflammation, with fewer side effects than traditional therapies [11,12]. The study was conducted in Alzheimer’s disease patients. The hippocampal tissues were isolated after postmortem, and cerebrospinal fluid was studied, which showed that there was a reduction in the levels of LXA_4_ in both the CSF and the hippocampus. Also, it was found that LXA4 and RvD1 levels in CSF correlated with Mini-Mental State Examination (MMSE) scores. It was concluded that a resolution pathway exists in the brain, and the alterations described herein strongly suggest a dysfunction of this pathway in AD. MMSE correlations suggest a connection with cognitive function in AD [13]. There are a number of animal studies that have shown the effectiveness of specialized pro-resolving mediators in decreasing inflammatory responses, along with promoting healing through tissue repair [14]. Crucially, resolvins also address the fundamental breakdown of resolution pathways that occur in many long-term inflammatory diseases. Since resolvins are a next-generation class of immune resolvers with both pro-resolving and anti-inflammatory properties, the therapeutic approach is shifting from simply suppressing inflammation to encouraging its physiological resolution. Bioactive compounds such as polyphenols, flavonoids, alkaloids, terpenoids, and omega-3 fatty acids modulate inflammation by inhibiting nuclear factor kappa B (“NF-κB”), MAPKs, and COX pathways, leading to reduced production of pro-inflammatory cytokines [15]. Many antioxidants act as scavengers of reactive oxygen species (ROS), thereby restoring redox balance. Others stimulate the production of specialized pro-resolving mediators (SPMs), which assist in both the prevention and resolution of inflammation. Due to their multi-targeted actions and low toxicity, bioactive compounds are emerging as effective adjuncts in managing both acute and chronic inflammatory diseases. Among many bioactive molecules found in plants, flavonoids like quercetin and rutin, and other significant bioactive molecules like resveratrol, glycyrrhizin, lycopene, and indole-3-carbinol are known to have been extensively studied for their antioxidant, anti-inflammatory, immunomodulatory, and cytoprotective properties, which suggest that they could be quite effective therapeutically in the treatment of chronic and metabolic diseases.

The creation of novel pharmaceutical molecules is a difficult and lengthy process, leading to the increased study of biologically active natural molecules present in plants, algae, seaweed, and microbes. Nevertheless, these molecules have several constraints, such as sensitivity to heat, inconsistency in the process of extraction, costly manufacturing, and loss of effectiveness after processing or storage. Furthermore, there can be differences in the effects of these biologically active molecules between different people because of differences in age, sex, lifestyle, metabolism, and absorption [16].

This paper aims to critically examine the impact of bioactive compounds on connected pathways of inflammation and resolution in chronic diseases. The paper discusses the latest developments in the field of anti-inflammatory bioactive compounds and therapeutic strategies that stimulate inflammation resolution processes, pointing out their mechanisms and effectiveness. Other trends, such as those involving gut microbiota metabolites, multifunctional plant compounds, targeted drug delivery using nanoencapsulation, and personalized resolution approaches, are examined in relation to their significance in optimizing treatment outcomes. The research areas that require further attention and development are also outlined, including poor translatability of research findings into practice, lack of human experiments, and absence of standardized dosing regimens. Moreover, the study considers an integrated approach to linking bioactive compounds with pro-resolving mechanisms (favorable tissue repair and regeneration) and anti-inflammatory actions.

An extensive literature search was carried out via PubMed, Scopus, Web of Science, Google Scholar, and ScienceDirect for research articles published from 2000 until 2026 on bioactive anti-inflammatory molecules, inflammation resolution, and chronic diseases. Search terms including “bioactive compounds,” “pro-resolving mediators,” “microbiome metabolites,” “nano-capsules,” “NF-κB pathway,” “Sirtuin-1,” “targeted drug delivery,” and “tissue repair and regeneration” were applied with Boolean operations. Peer-reviewed articles published in the English language in vitro, in vivo, preclinical, and clinical and mechanistic settings were included, while editorials, duplicate articles, conference proceedings, non-English articles, and insufficiently documented articles were excluded.

## 2. Bioactive Compounds in Inflammation Management

Chronic inflammation is a key pathological and physiological mechanism underlying a wide range of diseases, including metabolic syndromes such as diabetes and obesity, neurodegenerative disorders, cardiovascular disorders, and autoimmune disorders [17]. Inflammatory stimuli, such as dietary stress, can increase ROS and the redox-sensitive transcription factor NF-κB [18]. Dietary stress enhances the production and translocation of gut-derived lipopolysaccharide (LPS) into the systemic circulation, thereby activating TLR4 and NF-κB and elevating pro-inflammatory cytokine levels [19]. The production of pro-inflammatory cytokines, such as interleukins (ILs) and TNF-α, also contributes to the activation of the MAPK and NF-κB pathways [20]. Activation of transforming growth factor β-activated kinase 1 (TAK1) initiates MAPK signaling cascades, including extracellular signal-regulated kinases (ERKs), JNKs, p38, and MAPKs [21,22].

Bioactive compounds are non-essential molecules that have unique physiological or cellular effects on living organisms [23]. The unique chemical structures of these compounds make them good at quenching ROS and maintaining redox equilibrium [24]. Bioactive compounds, including astaxanthin, nicotinamide riboside, and resveratrol, can selectively activate SIRT1 [25]. Research has delineated the conventional functions of SIRT1 in the modulation of inflammation, metabolic diseases, apoptosis, and cell cycle regulation [26]. SIRT1 inhibits NF-κB-mediated TNF-α and inflammation by deacetylating its subunit, RelA/p65, thereby preventing NF-κB from doing its work [27]. SIRT1 inhibits the p300/CREB binding protein (p300/CBP), a key acetyltransferase, and prevents p65 acetylation, which is necessary to stop NF-κB-mediated gene expression [28]. The p300/CBP is known for helping to acetylate poly (ADP-ribose) polymerase 1 (PARP1), which is necessary for activating NF-κB [29].

The majority of the existing anti-inflammatory regimes have been found to be ineffective in tackling chronic conditions due to their inability to restore physiological functions. In view of this situation, there is a need for alternative treatment methods that can address the complexity of the inflammation process and help promote effective disease healing without any side effects.

Bioactive compounds existing in nature have gained considerable attention due to their capability to control crucial inflammatory signaling pathways implicated in chronic diseases. Bioactive compounds isolated from plants used for medicinal purposes, metabolites extracted from marine organisms, and bioactive compounds synthesized by gut microbes have exhibited tremendous promise in targeting signaling pathways like NF-κB, MAPK, cytokine cascades, and the NLRP3 inflammasome. These molecules operate on several molecular targets, which could prove useful in managing persistent inflammation related to various conditions, such as cancer, neurodegenerative ailments, and cardiovascular disorders. As compared to traditional synthetic drugs, several natural bioactive compounds exhibit reduced toxicity and improved biological compatibility, thus offering promising options for designing effective anti-inflammatory therapies.

Phenolics such as curcumin from Curcuma longa inhibit NF-κB translocation and COX-2 expression, thereby attenuating pro-inflammatory cytokines (TNF-α, IL-6, and IL-1β). Resveratrol, abundant in grapes and berries, activates SIRT1 to suppress MAPK signaling, reducing oxidative stress and leukocyte infiltration. Flavonoids such as quercetin (found in onions and apples) and epigallocatechin gallate from green tea also exhibit anti-inflammatory and antioxidant effects.

There exist several bioactive components, such as resveratrol, lignans, terpenoids, lycopene, glycyrrhizin, indoles, and indole-3-carbinol, which exhibit anti-inflammatory, antioxidative, and immunomodulatory properties in different in vitro and in vivo studies; yet, the magnitude of these effects depends greatly upon the concentration of the compound used, its formulation, the nature of the disease, and the time of exposure. Resveratrol has been found to suppress the activation of the NLRP3 inflammasome and prevent macrophage pyroptosis at concentrations usually lying in the range of 5 to 100 µM in vitro studies and 10 to 100 mg/kg in animals, while some studies have reported the ineffectiveness of this component because of its poor bioavailability and high metabolism. Lignans and terpenoids, including ginsenosides extracted from Panax ginseng, were also found to modulate JAK-STAT and NF-κB signaling pathways at doses ranging from 10 to 50 mg/kg in vivo experiments; however, differences in standardization of extracts and pharmacokinetics of these components have led to inconsistent results. Lycopene and glycyrrhizin exhibited antioxidative and hepatoprotective roles in some in vitro experiments; yet, low concentrations of these compounds could lead to the inability to affect the inflammatory responses effectively. In addition, indoles and indole-3-carbinol have shown their capacity to regulate inflammatory processes and apoptosis at higher concentrations in vitro; nevertheless, dose-dependent toxicity and inconsistent absorption characteristics pose problems in translating these components into clinical practice. Alkaloids such as berberine, which have been found in *Berberis* L. plants, have proved their ability to downregulate TLR4-mediated inflammation responses; nevertheless, their oral bioavailability poses challenges in using them for clinical applications [30]. Moreover, despite the possible benefits of combinations of polyphenols and nanotechnologies (e.g., liposome-based and nanoparticle-based formulations), little evidence exists to support their long-term safety and reproducibility [31]. Consequently, more standardized research and dose-optimization studies are needed before the application of these compounds in medicine. The details of representative in vitro and in vivo studies are summarized in Table 1 and Table 2.

### 2.1. Natural Plant Compounds Targeting the Inflammatory Cascade

The primary components of the Mediterranean diet include fruits, vegetables, and all plant-based foods. These foods are rich in phytochemicals (natural bioactive compounds) and antioxidant capabilities [48]. Natural bioactive compounds can be categorized into various groups according to their chemical structure and biological functions, such as phenolics, carotenoids, alkaloids, phytosterols, nitrogen-containing chemicals, and organosulfur compounds [49]. These compounds promote various beneficial health effects, including cardioprotective, anti-atherosclerotic, anticarcinogenic, bowel-protective, and neuroprotective effects [50]. Although the results presented in Table 1 and Table 2 demonstrate the medicinal application of these bioactive compounds, the effectiveness demonstrated can only be attained depending on the dosage employed, resulting in significant variations between the results presented.

#### 2.1.1. Polyphenols

Polyphenols are a broad class of phytochemicals found in many plants. These compounds are abundant in fruits and vegetables [51]. Researchers are particularly interested in them because they can combat inflammation, stimulate the immune system, and act as antioxidants [52,53].

Resveratrol (3,5,4′-trihydroxy-trans-stilbene) is a stilbene polyphenol found mostly in the skins of grapes, berries, and peanuts, with red wine being a major dietary source. Resveratrol exhibits potent anti-inflammatory, antiviral, antioxidative, and antibiotic activities [54]. Researchers have extensively studied its biological activity and have discovered that it plays an important role in modulating the body’s inflammatory response at the molecular level. Resveratrol’s properties can be categorized into two distinct groups. One function regulates the redox state, while the other controls histone acetylation. The former property aligns with its chemical structure, which features conjugated double bonds [55]. Resveratrol scavenges free radicals, thereby shifting the diet-induced oxidation state of the microenvironment toward a reduced state, which is associated with decreased oxidative stress [56]. The latter property pertains to its capacity to activate histone deacetylases (HDACs), including SIRT1, a crucial enzyme for resveratrol’s protective functions [57]. Two pathways of resveratrol have been demonstrated to inhibit inflammation. In a myofibroblast cell derived from the human colon, resveratrol diminished TNF-α-induced ROS production and increased SIRT1 activation [58]. Furthermore, it diminished TNF-α-induced activation of intercellular adhesion molecule-1 (ICAM-1), which contributes to inflammation independently of SIRT1, as demonstrated by EX-527, a SIRT1 inhibitor, and SIRT1 knockdown [59]. SIRT1 represses NF-κB through the deacetylation of the RelA/p65 subunit at lysine 310 [60]. Resveratrol collectively inhibited TNF-α-induced inflammation via redox and acetylation-regulated pathways [61].

Resveratrol’s protective role against lipid peroxidation in cell membranes and against DNA damage may derive from its capacity to scavenge or neutralize free radicals [62]. Resveratrol has beneficial effects on metabolic and cardiovascular illnesses, malignancies, tuberculosis, and several age-related diseases [63]. The various effects of resveratrol are linked to its ability to activate SIRT1 [64]. In mice infected with *Mycobacterium tuberculosis*, resveratrol inhibits TAK1, thereby preventing phosphorylation and ubiquitination that would otherwise inactivate related signaling pathways, including MAPK and NF-κB, by activating SIRT1 [65]. The TLR2-p38 pathway regulates SIRT1 production in peritoneal macrophages from mice and humans with active TB [65]. In mice with paraquat-induced lung injury, resveratrol reduces oxidative stress and lung damage by promoting substantial signaling between SIRT1 and NrF2. Resveratrol elevated SIRT1 expression in the lung tissue of mice exposed to paraquat and mitigated oxidative stress and lung damage via NrF2 activation [66]. The interplay between SIRT1 and Nrf2 in the context of resveratrol therapy appears to be complex. SIRT1 promotes NrF2 by reducing its acetylation, which increases under oxidative stress. The antioxidant properties of NrF2 may be influenced by its acetylation state, which is regulated by the balance between acetylation and deacetylation. Resveratrol activates SIRT1, which has been demonstrated to improve Nrf2-mediated gene expression by increasing nuclear translocation, DNA binding, and transcriptional activity [67]. Studies have demonstrated that increasing NrF2 acetylation reduces its stability, thereby reducing its ability to combat free radicals. Resveratrol deacetylates SIRT1 and enhances NrF2 stability in cells or tissues, as oxidative stress is associated with SIRT1 acetylation [68]. Resveratrol has been shown to reduce fibronectin and TGF-β1 production induced by advanced glycation end products in the kidneys of diabetic rats and in mesangial cells [69]. The efficacy of resveratrol is attributed to its pronounced antioxidant properties, mediated by the NrF2/ARE pathway induced by SIRT1 [70].

#### 2.1.2. Lignans

Lignans are diverse polyphenolic chemicals predominantly located in plant-based foods such as flaxseeds, sesame seeds, whole grains, legumes, and various vegetables. These compounds are also classified as phytoestrogens due to their structural similarity to estrogen and their ability to elicit minor estrogenic or anti-estrogenic effects in the human body [71]. Lignans are classified into seven categories, including secoisolaricirestinol, sesamin, pinoresinol, matairesinol, medioresinol, syringaresinol, and lariciresinol. Potent anti-inflammatory, antioxidative, anticancer, antifungal, and antibacterial properties have been reported among these lignans [72,73].

Gut bacteria chemically transform lignans, a process that underlies their biological activities [23]. Secoisolariciresinol diglucoside (SDG), a conjugated lignan present in flaxseed and sesame seeds, is not digested in its native form. Instead, human gut microbiota catalyze its deglycosylation, converting SDG into secoisolariciresinol [74]. *Peptostreptococcus productus* and *Eggerthella lenta* are two bacterial strains that can demethylate or dehydroxylate unconjugated secoisolariciresinol. This process yields the metabolites enterodiol and enterolactone, which are readily absorbed from the gastrointestinal tract [75]. Microbial digestion of extra lignans in the gastrointestinal tract may lead to the formation of enterodiol or enterolactone metabolites in the colon, which are bioactive and can modulate inflammatory responses.

Several cellular and molecular mechanisms are involved in inflammation. The Janus kinase (JAK)-signal transduction and activator of transcription (STAT) pathway is important for T-cell development and β-cell switching. It receives signals from both type I and type II cytokines. Phosphorylation and translocation of NF-κB and AP-1 subunits can turn on the NF-κB and MAPK pathways. This transcription factor increases the levels of pro-inflammatory cytokines, including TNF-α, IL-1β, IL-6, COX-2, and iNOS. Studies indicate that dietary lignans and metabolites produced by gut bacteria may help mitigate the inflammatory cascade. Studies indicate that secoisolariciresinol diglucoside inhibits the NF-κB pathway, thereby blocking activation of the NLRP1 inflammasome [76]. Oral administration of SDG increased serum enterolactone concentrations and ameliorated atopic dermatitis in a murine model by suppressing Th2-driven immune responses [77]. Elevated enterolactone suppressed the Th2 cell response by interfering with the JAK-STAT6 signaling pathway [77].

Lignans not only regulate NF-κB but also diminish oxidative stress and act as mediators of inflammation. They accomplish this by eliminating ROS and enhancing the activity of endogenous antioxidant enzymes such as superoxide dismutase (SOD) and glutathione peroxidase (GPx). This antioxidant function not only safeguards cellular components from oxidative damage but also inhibits the activation of redox-sensitive inflammatory pathways [78].

Hui Hui Xiao et al. found that the lignan-rich fraction of *Sambucus williamsii* increased HDL and LDL levels in an ovariectomized (OVX) mouse model, while concurrently reducing total cholesterol and triglyceride levels. The extract significantly reduced LDL levels in both serum and liver. The extract reduced serum cholesterol and triglycerides in OVX animals by decreasing cytokine levels, including TNF-α, IL-22, and MCP-1, and by modifying gut microbiota [79].

#### 2.1.3. Lycopene

Lycopene is a symmetrical non-provitamin tetraterpene that consists of 40 carbons, 56 hydrogens (C_40_H_56_), and an isoprene unit. It is a carotenoid that is primarily found in red fruits and vegetables, such as tomatoes, watermelon, papaya, asparagus, carrots, and pink grapefruit [80]. It has been studied extensively because it is a strong antioxidant, anticancer, and anti-inflammatory agent [81]. Its unique chemical structure, which includes a long chain of conjugated double bonds, enables it to effectively quench singlet oxygen and neutralize ROS [79]. This reduces oxidative stress, a primary trigger and amplifier of the inflammatory cascade. In addition to its antioxidant properties, lycopene exerts direct anti-inflammatory effects by modulating the expression of key signaling pathways [82].

One of the most important ways is the inhibition of the NF-κB pathway, which is a key controller of immunological and inflammatory responses [83]. In cardiovascular studies, lycopene has been demonstrated to decrease TNF-α-induced NF-κB activation, ICAM-1 expression, and endothelial cell–monocyte interactions [84]. Furthermore, lycopene suppresses T-lymphocyte activation and reduces macrophage metalloproteinase secretion [85]. Recently, lycopene has proven to be an effective antiglycation agent, lowering the synthesis of advanced glycation end products (AGEs) and AGE receptors (RAGE), thereby contributing to vascular protection [86].

In an animal study of pancreatic disease, lycopene was found to reduce the inflammatory cytokines TNF-α and IL-1β, as well as the enzyme myeloperoxidase, while increasing the antioxidant glutathione level [87]. Lycopene alters the breakdown of IκBα and the movement of NF-κB into the nucleus. This stops the transcription of pro-inflammatory genes, thereby lowering levels of cytokines such as TNF-α, IL-1β, and IL-6 [88]. Lycopene has been shown to modulate cyclooxygenase-2 (COX-2) activity, an enzyme responsible for the synthesis of pro-inflammatory prostaglandins, by inhibiting its activation [89]. This mechanism represents an additional pathway by which lycopene regulates the inflammatory cascade. Collectively, these actions not only attenuate acute inflammatory responses but also reduce chronic inflammation. Notably, lycopene bioavailability is strongly influenced by the food matrix, processing methods, and the presence of dietary lipids, with cooked tomato products and oil-based formulations markedly enhancing its absorption.

#### 2.1.4. Glycyrrhizin

Glycyrrhizin, a triterpenoid saponin isolated from the roots of *Glycyrrhiza glabra* (licorice), has been regarded in traditional medicine for its anti-inflammatory, immunomodulatory, antiviral, antibacterial, anticancer, and hepatoprotective activities [90]. Studies show that glycyrrhizin lowers oxidative stress and inflammation by blocking the Hmgb1 and NF-κB signaling pathways. This results in reduced levels of malondialdehyde (MDA) and cytokines (TNF-α, IL-1β, and IL-6) in lung cancer cells [37]. These anti-inflammatory actions primarily result from altering cytokine production and inhibiting NF-κB activation, an important transcription factor involved in inflammatory signaling pathways [91]. Glycyrrhizin controls the production of TNF-α, IL-6, iNOS, and MCP-1 in liver cells. It also increases glutathione-S-transferase (GST) levels and decreases MDA levels [92]. It inhibits the NF-κB pathway by obstructing TLR4 in renal cells and reducing intracellular ROS levels [93]. In vitro studies have demonstrated activation of the AMP/NrF2 signaling pathways, leading to upregulation of antioxidant enzymes, including HO-1, NQO-1, and GCLC, alongside suppression of pro-inflammatory mediators such as TNF-α, IL-1β, and IL-6 [94]. Concurrently, glycyrrhizin enhances the production of anti-inflammatory cytokines, notably IL-10, which plays a critical role in maintaining immune homeostasis and preventing excessive immune activation. Collectively, glycyrrhizin’s immunomodulatory properties underscore its potential as a therapeutic candidate for autoimmune and chronic inflammatory disorders.

#### 2.1.5. Indoles

Indoles are naturally occurring substances abundant in cruciferous vegetables such as broccoli, cabbage, and cauliflower. They have garnered significant attention due to their effectiveness in combating inflammation and free radicals. Data from a previous study suggest that indole-3-carbinol (I3C) can modify immune system function and reduce oxidative stress [95].

Scientific study reveals that RAW264.7 macrophages challenged with LPS and I3C significantly reduced the production of nitric oxide (NO), TNF-α, IL-6, and IL-10, suggesting a role in dampening innate immune responses [96]. In mice, I3C reduced acute lung injury by lowering IL-6, TNF-α, and IL-16 levels in bronchoalveolar lavage fluid and suppressing immune cell infiltration, primarily through modulation of TRIF-dependent signaling pathways [97]. Topical I3C reduced serum IgE levels, epidermal thickness, and scratching behavior in a mouse model of atopic dermatitis while downregulating key inflammatory mediators, including thymic stromal lymphopoietin (TSLP) and periostin [98]. Its efficacy extended to gastrointestinal inflammation, where I3C improved DSS-induced colitis by activating the aryl hydrocarbon receptor (AhR), increasing antimicrobial peptide production, and restoring mucosal integrity [99]. Notably, in TNBS-induced colitis, I3C demonstrated gender-specific effects, with considerable improvements in outcomes in female mice [100]. I3C also demonstrated neuroprotective effects in Parkinson’s disease and cerebral ischemia models, where it decreased microglial activation, reduced pro-inflammatory cytokines, and increased anti-inflammatory markers such as IL-4 and IL-10 [101]. Moreover, advanced delivery systems, including nanocapsules and hydrogels, have been shown to enhance the bioavailability and anti-inflammatory efficacy of indole-3-carbinol (I3C) in skin inflammation models [102]. Collectively, these findings indicate that indoles exert significant anti-inflammatory effects and may contribute to the attenuation of chronic inflammation, highlighting their therapeutic potential in conditions associated with sustained immune activation.

### 2.2. Marine-Derived Compound Targets the Inflammatory Cascade

Marine environments host a diverse array of organisms, such as cyanobacteria, macroalgae, and marine invertebrates. These organisms serve as valuable sources of bioactive chemicals with potent anti-inflammatory properties [17]. Marine-derived drugs influence various processes, including inhibiting key inflammatory enzymes (e.g., COX-2), altering immune cell function, and reducing oxidative stress pathways [103]. Cyanobacteria, often called blue-green algae, have attracted significant attention for their numerous beneficial properties [104]. Phycocyanin, a compound found abundantly in Spirulina species, is among the most extensively researched. It combats inflammation by inhibiting the NF-κB signaling pathway and reducing levels of pro-inflammatory cytokines such as TNF-α and IL-6 [105]. Phycocyanin reduces COX-2 expression and inhibits white blood cell migration to inflamed tissues. This is particularly beneficial for managing chronic inflammatory conditions such as rheumatoid arthritis and ulcerative colitis [106]. Macroalgae, including brown, red, and green varieties, produce polysaccharides such as fucan, fucoidan, carrageenan, and agar, which enhance immune function and reduce oxidative stress [107].

Fucans are sulfated polysaccharides with fucose backbones. Recent research suggests that some fucose residues contain 3-linked fucose with 4-sulfated groups. Sulfated fucans, such as carrageenans from red algae, fucoidans from brown algae, and ulvans from green algae, have numerous benefits, including coagulation regulation, anticancer, antidiabetic, antioxidant, antithrombotic, and anti-inflammatory characteristics [108]. Algae-derived soluble polysaccharides are being studied as potential prebiotics and dietary fiber for humans [109]. Fucoidan has been shown to inhibit NF-κB activation, thereby suppressing the release of inflammatory mediators. These algae extracts suppress COX-2 activity and interrupt inflammatory cells from getting into affected tissues, enhancing their effectiveness in treating inflammation-related diseases [110].

Seaweeds are another major source of sulfated polysaccharides and exhibit diverse biological activities. It has been observed that the architectures of these molecules differ among algal species [111]. Galactan, which is made up completely of galactose or its modified units, is the most common sulfated polysaccharide in red algae. Fucans, which are a group of sulfated L-fucose-based polydisperse molecules, make up most of the brown algae [112]. Ulvans are the most common water-soluble polysaccharides in green seaweed. Some types of ulvans are a major source of sulfated galactans in *Codium* species (Chlorophyta, green algae). Numerous studies have demonstrated that sulfated polysaccharides exhibit a wide range of biological activities, including anti-inflammatory, antioxidant, anti-adhesive, anti-proliferative, antiviral, anticancer, and anticoagulant effects [113]. In addition to these bioactivities, sulfated galactans are widely used in the food industry for their excellent thickening and gelling properties [114]. Some red seaweeds contain sulfated polysaccharides, such as xylomannan sulfate and carrageenan. Carrageenan is a linear sulfated polysaccharide made up of 3,6-anhydro-D-galactose and D-galactose. It comes from some types of red seaweed. Lambda, kappa, and iota carrageenans are the three main types [115]. However, seaweeds typically do not produce pure carrageenans; rather, they generate various hybrid structures.

Copolymers of iota- and kappa-carrageenan exhibit distinctive gelation behavior and are widely employed in food manufacturing due to their strong physicochemical properties, including effective stabilization, gelling, and thickening capabilities [116]. These copolymers are commonly found in food products such as jams, ice cream, puddings, and yogurt. Beyond their food applications, iota- and kappa-carrageenan copolymers are also utilized in pharmaceutical formulations, owing to their reported anti-inflammatory and anticoagulant activities [117].

### 2.3. Gut Microbiota Linkage with the Inflammatory Cascade

The gut microbiota is critical for regulating the immune system and reducing inflammation [118]. The gastrointestinal tract constitutes the largest mucosal surface area in humans, acting as a dynamic biologic gateway between the internal environment of the host and an array of environmental antigens. The interplay between these two environments is mediated through a sophisticated three-layer structure involving the physical barrier provided by the mucus, the highly specialized epithelial monolayer, and the immune system of the mucosa. Within the ecologic niche, the microbiota, composed of a dense network of bacteria, archaea, fungi, and viruses, serves as a “virtual endocrine organ,” whose role includes systemic secretion of bioactive compounds that regulate host metabolism, development of the immune system, and maintenance of the integrity of the intestinal barrier. In physiological states, a heterogeneous population of microorganisms ensures the release of anti-inflammatory mediators, such as SCFAs, which supply colonocytes with energy and support the expression of tight junction proteins, including claudin, occludin, and ZO-1. The tight barrier prevents the translocation of the luminal content into the blood circulation. Alterations in the composition, diversity, and function of such microbiota colonies lead to dysbiosis. Dysbiosis can be described as a situation where there is a loss in the abundance of good symbionts (such as *Bifidobacterium* and *Akkermansia*), while there is an excessive presence of pathogens, otherwise known as pathobionts, that survive in inflammatory conditions. A healthy and diverse microbial ecology promotes anti-inflammatory pathways, strengthens the epithelial barrier, and alters how different types of immune cells function [119]. The effects of dysbiosis do not remain confined to the gut alone; instead, there is a series of secondary responses that follow: 1. Barrier Damage: There is a reduction in mucus layer integrity and destruction of the tight junctions at the apex, a condition colloquially referred to as “leaky gut.” 2. Endotoxemia and LPS Translocation: The main cause of systemic inflammation, in this case, is the translocation of lipopolysaccharide (LPS), which forms the outer membrane of Gram-negative bacteria across the damaged mucosal lining into the blood. 3. Chronic Inflammatory Signaling Pathway: After entering the systemic circulation, the LPS molecule acts as a PAMP. It binds to TLR4, a receptor found on the surface of macrophages and other immune cells, activating the NF-κB pathway. This results in the continuous production of pro-inflammatory cytokines such as TNF-α, IL-1β, and IL-6, which have been linked to the development of a variety of inflammatory illnesses, including IBD, metabolic syndrome, rheumatoid arthritis, and neuroinflammatory syndrome [120].

**Figure 1:**
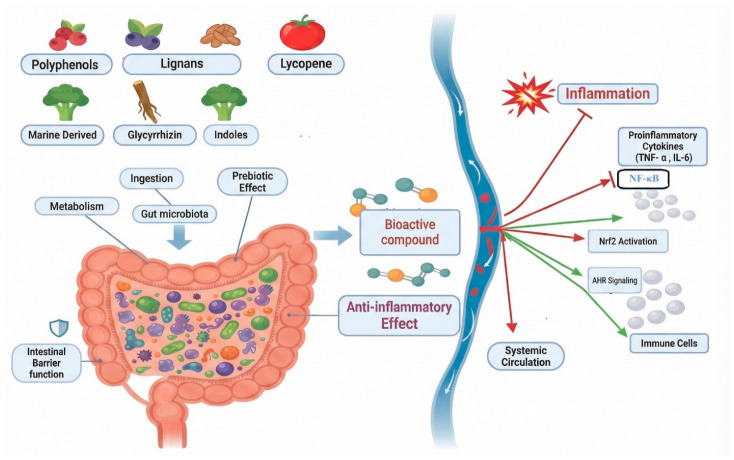
Conceptual model of dietary bioactive compound–gut microbiota interactions in anti-inflammatory signaling. Dietary bioactive compounds (polyphenols, lignans, lycopene, glycyrrhizin, indoles, and marine-derived compounds) reach the colon, where the gut microbiota convert them into bioactive metabolites (e.g., SCFAs, enterolactone, glycyrrhetinic acid) and promote beneficial microbial growth and gut barrier integrity. These metabolites enter the systemic circulation to suppress NF-κB–mediated pro-inflammatory cytokine production (red arrows), activate Nrf2-driven antioxidant responses (green arrows), and, for indoles, stimulate AhR signaling to maintain immune tolerance, collectively promoting inflammation resolution and host health.

The gut microbiota represents an important therapeutic target because it acts like a “virtual endocrine organ” controlling the processes of immune homeostasis and systemic metabolic turnover. One of the key mechanisms of this axis involves the mutual biotransformation of macromolecular polyphenols. Specifically, the high molecular weight flavonoids, such as rutin and quercetin, undergo hydrolytic cleavage by means of specific enzymes β-glucosidase and α-rhamnosidase, yielding phenolic acids characterized by a pronounced anti-inflammatory effect and able to limit the proliferation of LPS-producing pathobionts. Bioactive-driven modulation of the gut microbiota allows for selective colonization with resolving species, such as *Akkermansia muciniphila* and *Faecalibacterium prausnitzii*, ensuring the maintenance of the integrity of the intestinal mucosa and the prevention of the translocation of inflammatory mediators to the blood circulation. One of the primary ways that the gut microbiota affects the immune system is by producing fermentation-derived metabolites, particularly short-chain fatty acids (SCFAs) such as acetate, propionate, and butyrate. These SCFAs, which are produced by the anaerobic fermentation of dietary fibers, play a key role in the gut–immune axis [121]. SCFAs exert potent anti-inflammatory effects by modulating gene expression, cellular metabolism, and epigenetic regulation. Butyrate, for example, has been shown to inhibit histone deacetylases (HDACs), thereby increasing histone acetylation and reducing transcription of pro-inflammatory genes. SCFAs also affect the function of dendritic cells, macrophages, and T cells. They support regulatory T-cell (Treg) development and inhibit the production of inflammatory cytokines, including TNF-α and IL-6 [122]. As a result, the gut microbiota regulates immunological activation mostly through metabolic byproducts. The anti-inflammatory activities of SCFAs emphasize the importance of microbial fermentation in maintaining mucosal immunity and preventing chronic inflammation. Therapeutic interventions that restore microbial balance, such as prebiotics, probiotics, and dietary fiber supplements, have shown promise in treating dysbiosis-related inflammatory diseases and enhancing overall immunological resilience (Figure 1).

In order to further clarify the progression from experimental to clinical use, Table 3 shows an analysis of the pharmacokinetic challenges and current clinical standing of the major bioactive compounds involved in the anti-inflammatory process. The need to bridge the “bioavailability paradox” is clearly indicated by the table, which points out that it is necessary to translate the mechanisms of action found in experiments for human applications.

From the perspective of understanding NF-κB and SIRT1 as isolated signaling molecules, the conceptual framework for both molecules can now be seen as being linked via their antagonistic crosstalk, with energy metabolism and inflammatory response connected through them. SIRT1 is a longevity-related molecule, serving as a deacetylase enzyme involved in metabolic sensing, which reduces inflammation by repressing NF-κB, an inflammatory transcription factor. On the other hand, the persistent activation of NF-κB leads to a reduction in SIRT1, thus creating a vicious cycle [129,130].

The interaction between SIRT1 and NF-κB creates an intricate connection between inflammation and metabolism. SIRT1 blocks NF-κB by directly interacting with and deacetylating its p65 (RelA) subunit, reducing its transcriptional activity and pro-inflammatory cytokines such as TNF-α. However, when activated continuously, NF-κB promotes reactive oxygen species (ROS) generation and inflammatory microRNAs (miR-34a), thus causing the degradation of SIRT1 and metabolic dysfunction. SIRT1 promotes oxidative metabolism in mitochondria, whereas NF-κB directs cells towards glycolysis during inflammation. This imbalance leads to metaflammation [129,130,131,132].

Recently, more studies have uncovered the functions of the SIRT1-NF-κB axis in inflammation, metabolism, oxidative stress, and cell/tissue defense [129,130]. SIRT1 indirectly inhibits NF-κB through AMPK, which acts on IKKβ to inhibit p65 phosphorylation. Also, SIRT1 deacetylates the p300 coactivator, thereby inhibiting pro-inflammatory gene transcription, and protects mitochondria by regulating metabolism. In neurodegenerative diseases, the SIRT1 pathway reduces neuroinflammation. Resveratrol is an SIRT1 activator that suppresses microglial activity and provides neuron protection. Pharmacologically speaking, blocking the SIRT1-NF-κB loop defends against tissue injury (e.g., liver and kidney toxicity) [129,130,131,132].

## 3. Techniques to Promote Inflammation Resolution

### 3.1. Pharmacological Strategies

Multiple messengers are generated in a precise temporal and spatial manner throughout the inflammatory response, targeting specific receptors to prevent overshooting and maintain tissue homeostasis. Anti-inflammatory and pro-resolving therapeutic agents inhibit critical mechanisms of inflammation. Pro-resolving mediators, unlike anti-inflammatory agents, target specific processes for resolution: limiting or stopping neutrophil recruitment; promoting non-inflammatory monocyte recruitment; inducing neutrophil apoptosis; enhancing efferocytosis; reprogramming macrophages; returning healthy cells to the blood or lymphatic vasculature; and stimulating tissue healing and regeneration. Pro-resolving mediators include specialized pro-resolving lipid mediators (SPMs) such as lipoxins, resolvins, protectins, and maresins; proteins and peptides [annexin A1 (AnxA1), galectins, adrenocorticotropic hormone (ACTH), and IL-10]; gaseous mediators (H_2_S and CO); nucleotides (e.g., adenosine); and vagus-controlled neuromodulators. Below are some important pro-resolving mediators that help restore homeostasis after inflammation [133].

#### 3.1.1. Lipoxins

Pathogens are removed from the infection site by inflammation, which is followed by tissue homeostasis and inflammation resolution, thereby promoting successful host defense. Endogenous anti-inflammatory, pro-resolving lipoxins reduce chronic inflammation and tissue injury. Lipoxins are released by immunological cells, including neutrophils and macrophages. Lipoxins, a unique family of molecules having four conjugated double bonds, were originally identified from human immune cells by Serhan et al. Hamberg and Samuelsson extensively studied lipoxins, which are byproducts of the arachidonic acid pathway [134]. Multiple routes exist for the synthesis of lipoxins. Although lipoxin production is most commonly associated with cell–cell interactions, it can also occur in solitary cells. Inflammation, atherosclerosis, and thrombosis trigger rapid lipoxin production. During these processes, cell-to-cell interactions synthesize lipoxins. Transcellular biosynthetic pathways can be activated, leading to increased signaling by leukotrienes and prostaglandins, the generation of braking signals by novel substances, or both. Thus, lipoxin generation is a key step in the inflammatory response [135].

**Figure 2:**
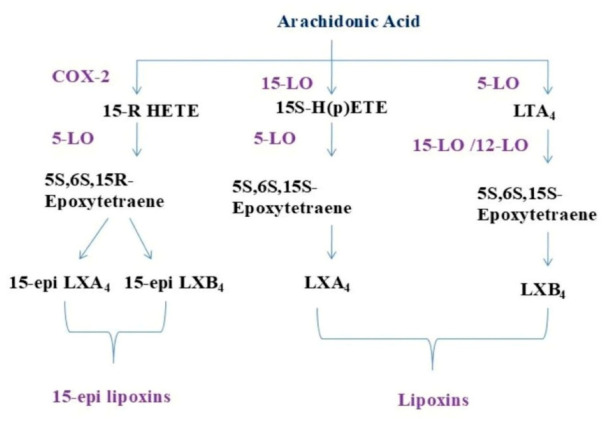
Biosynthesis of lipoxins. Adapted from [136] under Creative Commons Attribution 4.0 International License.

Lipoxin biosynthesis occurs primarily via three pathways, as shown in Figure 2. In the first mechanism, lipoxin synthesis requires the incorporation of molecular oxygen into the C_15_ position of arachidonic acid. Furthermore, 15-lipoxygenase (15-LO) is required for the production of lipoxins via this mechanism. Arachidonic acid is transformed to 15-hydroperoxyeicosatetraenoic acid (15-HPETE), a substrate for 5-LO in leukocytes, after being oxygenated at the C_15_ position. Hydrolases quickly convert this molecule to 5S,6R,15S-trihydroxy-7,9,13-trans-11-cis-eicosatetraenoic acid (LXA_4_) or 5S,14R,15S-trihydroxy-6,10,12-trans-8-cis-eicosatetraenoic acid (LXB_4_) by lipoxin B4 hydrolase. LXA_4_ and LXB_4_ promote vasodilation and control leukocyte activity [135].

The second mechanism of lipoxin production was identified through interactions between 5-LO, present in myeloid cells, and 12-LO, present in platelets. The 5-LO product, leukotriene A_4_ (LTA_4_), is rapidly taken up by platelets and converted to lipoxins via a 12-LO-dependent pathway. In both intracellular and transcellular eicosanoid synthesis, LTA4 acts as a mediator, since more than 50% is released from the original cell. During the interaction and co-activation of neutrophils and platelets, LTA_4_ can undergo various enzymatic and non-enzymatic conversions. These include LXA_4_ and LTB_4_ conversion by 12-LO; non-enzymatic hydrolysis (which happens in seconds in an aqueous environment); LTB_4_ conversion to LTC_4_ (a slow-reacting substance of anaphylaxis) by LTA_4_ hydrolase; and LTC_4_ conversion to LTC_4_ (a rapidly reacting substance of anaphylaxis) by LTC_4_ synthase. Cellular responses are highly dependent on the ratio of leukotriene to lipoxin formation, since LTB_4_ and LTC_4_ are potent pro-inflammatory mediators, and lipoxins inhibit leukotriene-mediated responses in living organisms [136]. 

Aspirin triggers lipoxin production through a distinct COX-2/5-LO pathway. Pro-inflammatory stimuli, including cytokines, bacterial infection, and hypoxia, induce COX-2 expression in endothelial and epithelial cells. Aspirin acetylates COX-2, redirecting arachidonic acid (C20:4) metabolism from prostanoid synthesis toward the generation of 15R-hydroxyeicosatetraenoic acid (15R-HETE). This intermediate is subsequently converted via transcellular interactions by leukocyte 5-LO into 15-epimer lipoxins, also known as aspirin-triggered lipoxins (ATLs) [136].

#### 3.1.2. Resolvins

**Figure 3:**
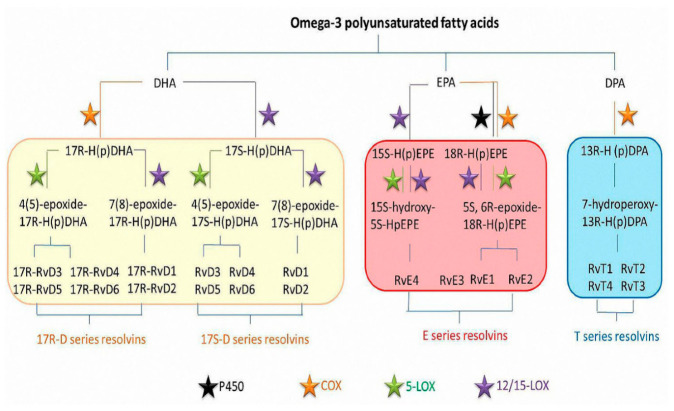
Biosynthesis of D, E, and T series resolvins. Reproduced from [15] under Creative Commons Attribution 4.0 International License.

LOXs, CYP450s, and COXs catalyze the metabolism of ω-3 polyunsaturated fatty acids docosahexaenoic acid (DHA), eicosapentaenoic acid (EPA), and docosapentaenoic acid (DPA) to produce resolvins. The D series resolvins RvD1 to RvD6 have been synthesized from DHA; the E series resolvins RvE1 to RvE4 and 18S-RvE1 have been synthesized from EPA; and the T series resolvins RvT1 to RvT4 have been synthesized from DPA. Figure 3 shows the synthesis of the D, E, and T series of resolvins [15]. Aspirin can also generate AT-series resolvins via the COX-2 pathway. Strong evidence suggests that resolvins such as RvE1, RvD1, and aspirin-triggered RvD1 (AT-RvD1) effectively reduce inflammation by promoting resolution and alleviating pain. Resolvins’ analgesic actions are mediated by GPCRs (e.g., ChemR23, GPR-32), expressed by immune cells, glial cells, and neurons. Resolvins reduce aberrant pain by lowering inflammation, glial activation, and spinal cord synaptic plasticity while maintaining normal pain experience. RvE1 inhibits the ERK signaling pathway, which regulates peripheral, central, and glial activation in the dorsal root ganglia and spinal cord. Resolvins suppress the synthesis of pro-inflammatory mediators by inhibiting the NF-κB pathway [137,138]. RvD2 also improves M2 polarization and lowers pro-inflammatory markers. One animal investigation demonstrated that long-term aspirin-triggered RvD1 therapy does not modify macrophage polarization in smoke-induced lung inflammation. Long-term AT-RvD1 treatment does not produce tissue fibrosis. RvD1 may affect macrophage polarization depending on the type of inflammation (acute or chronic) or the duration of resolvin exposure [138]. By altering immunological and glial cell activity, resolvins reduce inflammatory and neuropathic pain and nervous system inflammation [139].

#### 3.1.3. Protectins

Protectins are anti-inflammatory lipid mediators derived from DHA and characterized by a conjugated triene structure. Also termed neuro-protectins, due to their biosynthesis and actions in neural tissues, protectins inhibit polymorphonuclear neutrophil (PMN) infiltration, as do resolvins. Produced primarily by glial cells, these mediators suppress pro-inflammatory cytokine expression and contribute to inflammation resolution [140].

Various cell types, including murine brain cells, human microglial cells, human peripheral blood mononuclear cells, and Th2 CD4+ T cells, produce protectin D1 (PD-1)/neuroprotection D1 (NPD-1). In response to oxidative stress, retinal pigment epithelium cells produce NPD1 to avoid apoptosis. In vitro and in vivo, PD-1/NPD1 exhibits strong, stereospecific immunoregulatory functions, including anti-inflammatory and homeostatic effects, similar to those of resolvins. PD-1 supports retinal epithelial cell protection, ameliorates stroke-related ischemia–reperfusion injury, and shows benefits in animal models of Alzheimer’s disease (AD). In AD, the hippocampus shows specific decreases in DHA, NPD1, and ALOX15 levels, indicating dysregulation of neuroprotection. Additionally, NPD1 inhibits pro-inflammatory gene expression and leukocyte accumulation following an ischemic stroke. Specific PD-1 activities on T cells reduce migration, cytokine release, and cell survival [141,142,143].

#### 3.1.4. Maresins

Maresins are unique lipids synthesized from omega-3 fatty acids, which play an important role in promoting the resolution of inflammation in order to accelerate healing processes while curbing excess inflammation response. This family includes maresin 1 (7,14-dihydroxydocosa-4Z,8,10,12,16Z,19Z-hexaenoic acid, MaR1), maresin 2 (13R,14S-dihydroxy-docosahexaenoic acid, MaR2), and maresin conjugates in tissue regeneration (MCTR). Maresins have been detected in numerous human tissue fluids, and human macrophages can make MaR1. This process involves the conversion of DHA to 14S-HpDHA, with 12-lipoxygenase (12-LOX) being the major enzyme involved. Subsequently, the epoxidation of 14S-HpDHA leads to the synthesis of 13S,14S-epoxy-maresin. Finally, hydrolysis of 13S,14S-epoxy-maresin takes place to complete the process. Interestingly, the level of 12-LOX has been shown to be highest in M2 macrophages and dendritic cells. MaR1 can reduce polymorphonuclear leukocyte (PMN) infiltration in zymosan-induced acute peritonitis, promote macrophage phagocytosis of apoptotic PMNs and efferocytosis, accelerate tissue regeneration in planaria (MaR1 optimal dose: 100 nM), and inhibit pain. It modulates human phagocytes through the leucine-rich repeat-containing G protein-coupled receptor 6 (LGR6) pathway to trigger the pro-resolving phase of inflammation. Additionally, MaR2 and maresin conjugates in tissue regeneration (MCTR) have also been reported to be bioactive [144,145,146].

### 3.2. Therapeutic Implications of Lipoxins, Resolvins, Protectins, and Maresins

Inflammation is now recognized as a cause of many human diseases. Unbalanced pro-inflammatory and anti-inflammatory signaling systems, as well as altered expression of pro-resolving mediators, are linked to the development of inflammatory diseases, such as pre-eclampsia, and chronic inflammatory processes, including cardiovascular, respiratory, and neurological diseases [147,148]. Multicellular reactions during cell–cell interactions and single-cell responses create LXs in vivo. During inflammation, platelet–leukocyte interactions generate lipoxins A4 and B4, which function as “stop signals” to limit polymorphonuclear leukocyte recruitment at sites of inflammation and reperfusion injury. Genetic polymorphisms affecting specialized pro-resolving mediator (SPM) biosynthesis, receptor expression or function, or reduced fatty acid availability can impair lipoxin synthesis. Deficient local LXA_4_ production has been reported in atherosclerotic vessels, colonic mucosa of inflammatory bowel disease patients, gingival crevicular fluid of smokers, brain tissue of Alzheimer’s disease patients, alveolar macrophages in mild asthma, and bronchoalveolar lavage fluid from severe asthma patients [149].

Alzheimer’s disease starts with neuroinflammation. Mutations in microglial and innate immune cell genes promote activity and inflammation. Due to Toll-like receptor malfunction, chronic brain inflammation may impair microglial phagocytosis. Microglial apoptosis leads to Aβ accumulation and plaque formation; pro-inflammatory markers such as IL-1, IL-6, and TNF-α also promote neuroinflammation, recruit peripheral macrophages, and increase APP, ROS, and chemokine release. Additionally, there is evidence that specialized pro-resolving mediators (SPMs) and pro-inflammatory lipid mediators are imbalanced in the persistently pro-inflammatory brains of patients with AD, thereby worsening the disease. Consistent with this perspective, multiple animal models and studies in patients with AD have revealed sustained alterations in SPM levels [150,151]. Kantarci et al. found that transgenic 5xFAD mice had significantly lower hippocampal levels of RvE1, RvD2, and LXA_4_ than non-transgenic controls [152]. Dunn et al. found that LXA_4_ levels in the brain tissue of transgenic 3xTg-AD animals decline with age. SPMs biochemically promote inflammation resolution [153]. It appears that Alzheimer’s disease alters the pro-resolution pathway by lowering MaR1, RvD5, and NPD1. Inflammation resolution may slow cognitive decline in this illness. In a mouse neuron–microglia co-culture model, maresin-1 (MaR1) attenuated Aβ_42_-induced pro-inflammatory responses, including microglial chemotaxis and the production of TNF-α and IL-1β [150,151,154]. It has been proposed that reduced MaR1 levels in the entorhinal cortex and hippocampus of patients with Alzheimer’s disease (AD) prolong neuroinflammatory signaling. Consistently, postmortem hippocampal tissue and cerebrospinal fluid from AD patients exhibit decreased levels of lipoxin A4 (LXA_4_). Sustained neuroinflammation may upregulate glial ALX/FPR2 expression, thereby increasing tissue sensitivity to pro-inflammatory ligands such as Aβ peptides. Under these conditions, LXA_4_ deficiency may contribute to persistent, unresolved inflammation [150,151].

Thus, in AD, increasing LXs synthesis in the brain may reduce inflammation and improve outcomes, as neurons and glia express ALXR. Research indicates that LXA_4_ levels in AD patients’ brains are lower than in healthy controls, suggesting that poor resolution mechanisms may aggravate AD-associated neuroinflammation. Treatment with LXs and their derivatives, through IL-10- and TGF-β-mediated anti-inflammatory pathways, enhanced phagocytic clearance of β-amyloid and phosphorylated Tau (p-Tau) aggregates in human microglial cells and in mouse models of AD. The benefits of LX therapy may be due to reduced NF-κB/IL-1β pathway activity, which reduces p38, ERK, JNK, and GSK3β kinases involved in neuroinflammation and Tau phosphorylation [155].

Resolvin D4 (RvD4) levels in patients with AD are inversely associated with neurofibrillary tangle biomarkers and positively correlated with cognitive performance. Lipidomic analysis in an amyloid precursor protein (APP) knock-in mouse model revealed that age-related changes in brain lipid mediators (LMs) are more pronounced than amyloid-driven alterations, with the greatest increases in LM levels observed in aged animals despite early amyloid-β pathology. These findings suggest that lipid dysregulation may serve as an early indicator of pathogenic membrane remodeling in AD [156]. Consistently, postmortem analyses have shown reduced levels of DHA-derived neuroprotectin D1 (NPD1) in the CA1 region of the hippocampus in patients with AD, whereas levels in the thalamus and occipital lobes remain unaffected. Cerebrospinal fluid (CSF) lipidomics studies spanning the cognitive impairment continuum further demonstrate significant reductions in specialized pro-resolving mediators (SPMs), including RvD4, RvD1, PD1, MaR1, and RvE4, in AD and mild cognitive impairment compared with controls. Notably, RvD1 levels inversely correlate with phosphorylated Tau, whereas RvD4 levels inversely associate with neurofibrillary tangle burden, and both SPMs and their precursor fatty acids correlate positively with cognitive scores [157]. These findings support a neuroprotective role for SPMs in AD through the resolution of neuroinflammation, modulation of pro-inflammatory gene expression, and regulation of immune cell function [158]. Also, dietary n-3-derived SPMs can improve microglial energy metabolism and amyloid-β clearance by promoting a lipid-mediated switch from pro-inflammatory to pro-resolving SPMs (e.g., upregulating PPAR). This may be due to increased Sirt1 levels, which reduce NF-κB signaling and increase mitochondrial respiration [159].

Parkinson’s disease (PD) is a neurodegenerative disorder characterized by the loss of dopaminergic (DA) neurons in the substantia nigra (SN). Clinical signs include tremor, stiffness, and bradykinesia. Histologically, the presence of α-synuclein (α-syn)-rich Lewy bodies in CNS neurons is the hallmark of the disease [158]. Both experimental and clinical research indicate that oxidative stress and neuroinflammation play important roles in PD development. Inflammatory mediators contribute to DA neuron injury, while accumulated α-synuclein activates microglia and amplifies pro-inflammatory cytokine release, thereby exacerbating PD pathology. Recent studies demonstrate that several bioactive compounds can mitigate these pathological processes in PD models [160]. In particular, specialized pro-resolving mediators (SPMs) may confer neuroprotection by limiting chronic neuroinflammation and preserving the integrity of DA neurons [160]. Xu et al. demonstrated that DHA-derived specialized pro-resolving mediators (SPMs) attenuate pro-inflammatory responses in LPS-stimulated murine microglial cells [161]. Pretreatment with resolvin D1 (RvD1) significantly reduced nitric oxide (NO), TNF-α, and IL-1β production, as well as TNF-α, IL-1β, and inducible nitric oxide synthase (iNOS) mRNA expression, as determined by ELISA and RT-PCR. These effects were mediated through inhibition of MAPK signaling pathways, including ERK1/2 and p38 MAPK, and suppression of NF-κB and AP-1 DNA-binding activity. In a subsequent study, Xu et al. (2017) further demonstrated that RvD1 mitigates disease progression in PD models [161]. In vitro, RvD1 pretreatment (100–200 nM) dose-dependently reduced MPP^+^-induced apoptosis and cellular damage in PC12 cells, as shown by FITC and MTT assays. Consistent with microglial findings, RvD1 decreased TNF-α levels and inhibited p-p38, p-ERK, and NF-κB p50 signaling, supporting its anti-inflammatory and neuroprotective roles in PD models [161,162].

α-Synuclein (α-syn), a central pathogenic factor in PD, has been extensively investigated for its accumulation and neurotoxic effects. A recent study evaluated the impact of DHA-derived specialized pro-resolving mediators (SPMs) in a bacterial artificial chromosome (BAC) transgenic rat model overexpressing human α-syn. These animals displayed hallmark PD-like features, including enhanced dopaminergic neuron loss, reduced striatal dopamine release, and motor deficits, accompanied by pronounced neuroinflammation characterized by elevated cytokine production and microglial activation in the midbrain, striatum, and hippocampus [162]. To determine whether these neuroinflammatory alterations were associated with impaired inflammation resolution, levels of resolvin D1 (RvD1) and resolvin D2 (RvD2) were quantified in the cerebrospinal fluid and plasma of Syn rats [162]. Krashia et al. investigated the effects of resolvin D1 (RvD1) and resolvin D2 (RvD2) in plasma and cerebrospinal fluid (CSF) from Parkinson’s disease (PD) model rats and patients with early-stage PD. The rat model overexpressed human α-synuclein (α-syn). In these α-syn transgenic rats, CSF RvD1 levels declined progressively with age, with marked reductions at 2, 4, and 18 months compared with wild-type controls [163]. Consistently, patients with early-stage PD exhibited significantly decreased RvD1 levels in both plasma and CSF, indicating that RvD1 biosynthesis is impaired during PD progression [160,163]. The GPR37 gene is upregulated in the substantia nigra of patients with sporadic Parkinson’s disease (PD), and elevated levels of ecto-GPR37 peptides are detected in the cerebrospinal fluid (CSF) of PD patients but not in those with Alzheimer’s disease, suggesting ecto-GPR37 as a potential PD-specific biomarker. Tian et al. investigated the effects of intrathecal administration of resolvin D2 (RvD2) on neuroinflammation in an LPS-induced PD rat model in vivo and in primary microglial cells in vitro [150]. LPS stimulation of the substantia nigra pars compacta (SNpc) increased nitric oxide (NO), iNOS, pro-inflammatory cytokines (TNF-α, IL-1α, IL-1β, IL-6, IL-18), and ROS, accompanied by NF-κB p65 nuclear translocation and upregulation of IκBα and IKKβ. RvD2 treatment attenuated these inflammatory responses, inhibited TLR4/NF-κB pathway activation, and prevented behavioral deficits. These findings indicate that specialized pro-resolving mediators (SPMs) predict PD onset and progression and exert neuroprotective effects by suppressing inflammatory cytokine production, modulating signaling pathways, and improving behavioral outcomes [164,165].

In humans, susceptible atherosclerotic plaques are prone to acute atherothrombotic events, such as myocardial infarction and stroke, and are characterized by heightened inflammation and oxidative stress, extensive necrotic cores composed of uncleared apoptotic cells, and a thin collagen cap, a hallmark of defective inflammation resolution. We recently demonstrated that vulnerable regions of human carotid plaques exhibit a pronounced imbalance favoring pro-inflammatory lipid mediators, such as leukotrienes, over specialized pro-resolving mediators (SPMs), compared with stable plaque regions. Similarly, in Ldlr−/− and ApoE−/− mice fed a high-fat, high-cholesterol diet, advanced plaques displayed a marked reduction in the SPM-to-pro-inflammatory mediator ratio relative to early lesions. Causality has been supported across multiple animal models, where administration of SPMs (RvD1, RvD2, MaR1, or aspirin-triggered lipoxin A4) during advanced disease stages consistently slowed atherosclerosis progression and enhanced plaque stability. Notably, RvE1 reduced atherosclerosis progression in rabbits, an effect further potentiated by atorvastatin, while RvD1, RvD2, MaR1, and aspirin-triggered lipoxin A4 promoted lesional efferocytosis and plaque stabilization in mice. In ApoE−/− mice, annexin A1 similarly attenuated atherogenesis by regulating leukocyte trafficking via the ALX/FPR2 receptor. These findings demonstrate that impaired resolution signaling contributes to plaque vulnerability and that SPMs broadly limit atherosclerosis progression while promoting plaque stability [147].

Asthma causes chronic airway inflammation. Airway hyperresponsiveness, reversible bronchoconstriction, and remodeling are features of asthma. LXs reduce asthma, bronchoconstriction, and inflammation. LXA_4_ and its analogs have previously been shown to inhibit Cys-LT-mediated airway obstruction. They also stop LTB4-induced neutrophil and eosinophil chemotaxis. LXs also suppress granulocyte activation and block pro-inflammatory cytokines and chemokines, including T-lymphocyte cytokines, like corticosteroids. LXs may also promote NK cell-mediated death of eosinophils and neutrophils. Dufon et al. found that ALX/FPR2 KO mice have poor resolution [166]. In asthmatics with prolonged inflammation, ALX/FPR2 and pro-resolution agonists are absent. The mechanisms governing ALX/FPR2 expression can enhance endogenous anti-inflammatory responses. Anti-inflammatory qualities make LXs possible endogenous “braking signals” in the inflammatory process, which could help treat asthma [167].

Acute inflammation and infections generate bioactive lipids such as prostaglandins (PGs), leukotrienes (LTs), and thromboxane A2 (TXA_2_), as well as anti-inflammatory mediators such as LXs, protectins, maresins, and resolvins derived from arachidonic acid (AA). These bioactive lipid mediators increase macrophage phagocytic activity, reduce inflammation, and remove microorganisms. Lipoxins (LXs) and AA-derived bioactive lipid mediators can modulate SARS-CoV-2 infection by inhibiting viral entry and replication, downregulating ACE2 expression, and suppressing the production of pro-inflammatory cytokines [168]. Consequently, deficiencies in AA and pro-resolving lipid mediators may increase susceptibility to viral infections by impairing anti-inflammatory and resolution pathways. Pal et al. found that obese patients with fewer SPMs are more likely to contract SARS-CoV-2. Thus, oral or intravenous AA and LXs may increase SARS-CoV-2 resistance and recovery in COVID-19 [169]. Lee proposed that specialized pro-resolving mediators (SPMs), particularly lipoxins (LXs), may be effective in treating SARS-CoV-2 infection by modulating viral–inflammatory signaling circuits [170]. Consistently, Regidor et al. reported that SPMs attenuate disease severity and associated complications, including acute lung injury, immune thrombosis, and cytokine storm, primarily by suppressing pro-inflammatory cytokine production [171]. Hammock et al. further suggested that cytokine release during SARS-CoV-2 infection induces endoplasmic reticulum stress and an eicosanoid storm, which may be mitigated by enhancing anti-inflammatory LX pathways [172].

**Figure 4:**
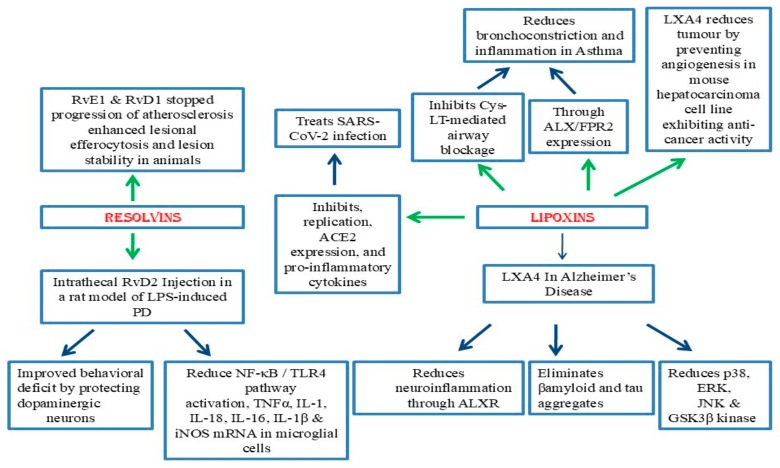
Therapeutic implications of lipoxins and resolvins in various diseases. Illustrations elaborate on the diverse biological functions of specialized pro-resolving mediators, specifically resolvins (RvE1, RvD1, and RvD2) and lipoxins (LXA4), in a range of disease models, including inflammatory, neurological, cardiovascular, respiratory, infectious, and oncological conditions.

Polyunsaturated fatty acids (PUFAs), including γ-linolenic acid (GLA), AA, EPA, and DHA, may exert anticancer effects by serving as precursors for lipoxins (LXs), resolvins, protectins, and nitro-lipids and by enhancing their biosynthesis [173]. Experimental evidence shows that lipoxin A4 (LXA_4_) suppresses vascular endothelial growth factor (VEGF) production and hypoxia-inducible factor-1α (HIF-1α) expression in mouse hepatocarcinoma (H22) cells [174]. Moreover, BML-111, a stable LXA_4_ analog, significantly reduced epithelial–mesenchymal transition (EMT) and migration in CoCl_2_-stimulated MCF-7 cells, according to in vitro tests. These effects were achieved by inhibiting *MMP-2* and *MMP-9,* which are downregulated by 5-lipoxygenase (5-LOX). Additionally, in BALB/c nude mice injected with MCF-7 cells, BML-111 prevented EMT and migration of breast cancer cells, which suggests that inhibiting the 5-LOX pathway may be a viable strategy for identifying useful therapeutic targets and that BML-111 may be a suitable therapeutic medication for breast cancer [175]. Collectively, these findings indicate that LXA_4_ limits tumor progression primarily by inhibiting angiogenesis. LXs, as endogenous anti-inflammatory mediators, may suppress carcinogenesis, tumor progression, metastasis, and tumor angiogenesis by resolving inflammation [173]. Figure 4 represents the therapeutic implications of lipoxins and resolvins in various diseases.

## 4. Challenges and Future Perspectives

### 4.1. Nanoencapsulation of Bioactive Compounds: Bioavailability Enhancement, Challenges, and Recent Advances

**Figure 5:**
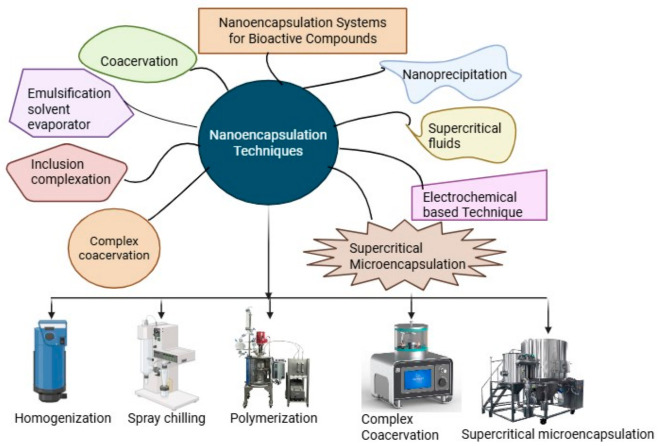
Schematic representation of major nanoencapsulation techniques used to enhance the stability, protection, and bioavailability of bioactive compounds. This figure illustrates various nanoencapsulation approaches employed to protect and enhance the delivery of bioactive compounds. Common techniques include nanoemulsification, nanoprecipitation, spray drying, coacervation, liposomal entrapment, and polymeric nanoparticle formation. Each method encapsulates sensitive bioactive compounds within nanocarriers, such as liposomes, solid lipid nanoparticles, nanospheres, or nanocapsules, thereby improving solubility, enabling controlled release, and enhancing resistance to degradation. These strategies collectively enhance the functional performance of bioactive compounds in food, nutraceutical, and pharmaceutical formulations.

It is becoming increasingly popular to use nanoencapsulation techniques in order to enhance the stability, delivery, and absorption of bioactive compounds. Nanoencapsulation allows bioactive molecules to be shielded against environmental factors and degradation by the gastrointestinal tract. Various methods of nanoencapsulation include nanoemulsification, formation of nanoliposomes, nanoprecipitation, electrospinning, electrospraying, and microfluidization, all of which can contribute to the physicochemical stability and bioavailability of the encapsulated bioactive compounds, as illustrated in Figure 5 [176].

The nanometric reduction in size results in an increase in the surface area/volume ratio, which facilitates dissolution, distribution, and absorption of the substance. Several nanocarriers like lipid nanoparticles, polymeric nanoparticles, nanoemulsions, and combined particles have displayed great potential in protecting compounds like curcumin and resveratrol against oxidation, enzymatic decomposition, and extreme gut environments and, at the same time, releasing the substances in a sustained manner [177,178]. Nanoencapsulation also helps in improving storage stability due to less degradation by light, variable pH, and oxidation [179]. Some studies have indicated the possibility of nanoparticle passage through the intestinal membrane via endocytosis, paracellular transport, and mucoadhesion [180].

Nevertheless, the delivery of bioactive molecules, including polyphenols, peptides, vitamins, and hormones, is challenging since several of these biomolecules are characterized by low water solubility, poor permeability, high metabolic activity, and digestive instability [181,182]. The application of nanoencapsulation in this regard facilitates overcoming the mentioned constraints through ensuring enhanced dispersibility, protection from degradation, and improved bioavailability [183].

The choice of the particular nanocarrier system plays an important role in the successful encapsulation. The use of lipids, including liposomes, solid lipid nanoparticles, and nanostructured lipid carriers, is preferable due to biocompatibility and protection against the destruction of substances during transport. Nevertheless, problems such as inefficient encapsulation, aggregation of particles, and leakage during storage make it difficult to apply the above systems [184,185]. Nanofibers, on the other hand, have benefits such as controlled delivery and targeting of substances, yet their preparation requires complicated conditions [176,186]. An additional important parameter in nanoencapsulation technology is the difference between bioaccessibility and bioavailability. The term bioaccessibility defines the liberation of the bioactive compound from the carrier in the gastrointestinal tract, while the term bioavailability indicates the amount of compound available in a biologically active state in the bloodstream [186]. Many parameters, like digestive enzymes, stomach environment, dietary content, and even gut flora, can impact this process. Nanoencapsulation increases bioaccessibility through enhanced solubility and protection of compounds till they reach their target site for absorption.

Recent advancements in nanoencapsulation include multifunctional and biomimicking carrier systems. The use of casein micelles for the encapsulation of nutrients has been successful due to their ability to protect the nutrients and improve the stability of the formulation in the gastrointestinal tract by means of sub-micellar calcium phosphate structures [187]. Similar is the case with NLC formulations that contain both solid and liquid lipids, which offer improved loading capacity, chemical stability, and minimal leakage losses during storage [188].

Current studies are increasingly focused on intelligent nanocarriers that release the encapsulated compounds in response to changes in pH levels, temperature, and enzymes [179,180,184,189]. Systems of co-encapsulation for delivering more than one bioactive compound at a time are now gaining popularity due to their synergistic effect [187]. Moreover, modern techniques like nanospray drying, electrospinning, electrospraying, and supercritical fluids are making the process more efficient and stable for encapsulating delicate phytochemicals and essential oils [190,191]. Furthermore, nanostructured lipid carriers and solid lipid nanoparticles are being considered as potential candidates for encapsulating marine bioactive compounds to enhance their oral bioavailability [192]. Overall, it can be seen that nanoencapsulation technology has a promising future in terms of enhancing the functionality and performance of bioactive compounds.

### 4.2. Hybrid and Combinatorial Platform for Bioactive Delivery Systems

Combinatorial delivery systems integrate multiple carrier platforms to synergistically enhance the stability, bioavailability, and controlled release of bioactive compounds. These hybrid systems leverage the complementary strengths of different nanocarriers to overcome the inherent limitations of single-component formulations. Lipid–polymer hybrid nanoparticles (LPHNs) are perhaps the most popular type of such systems. They comprise a hydrophobic polymer core covered with either a lipid monolayer or bilayer. The use of this structure allows one to benefit from the high mechanical strength provided by polymer structures, as well as from the biomimetic properties of lipids. In the case of anti-inflammatory treatment, LPHNs are created specifically to deliver curcumin and resveratrol, thereby significantly lowering the rate of initial burst and increasing the half-lives of these drugs in systemic circulation. Another example of combinatorial systems is provided by liposome–polymer hybrids; they include coatings made of biopolymers such as chitosan or PEG that provide steric stabilization and mucoadhesive properties and thus increase the residence times of active substances in the GI track or on the mucosal surface. Lipid–polymer hybrid nanoparticles, liposome–polymer hybrids, lipid-based nanocarriers (SLNs and NLCs), and multi-stage nanocarriers represent advanced combinatorial nanoencapsulation systems designed to overcome stability, solubility, and controlled-release limitations of bioactive compounds. Lipid–polymer hybrid nanoparticles combine the stability of a polymeric core with the biocompatibility of a lipid shell, enabling enhanced drug loading, reduced burst release, and improved physiological stability, as demonstrated with compounds such as curcumin and resveratrol [193,194,195]. Similarly, liposome–polymer hybrid systems improve liposomal stability and enable controlled release of both hydrophilic and hydrophobic compounds, while biopolymer surface modifications enhance mucoadhesion and targeting potential [196]. Lipid-based nanocarriers, including SLNs and NLCs, provide protection and sustained release for lipophilic compounds, with NLCs offering superior drug loading and storage stability due to their mixed lipid matrix [197]. Multi-stage or layered nanocarriers further enhance therapeutic efficacy by enabling stimulus-responsive, sequential payload release, thereby improving peptide stability and bioavailability [198]. Other combinatorial approaches, such as nanoemulsion–hydrogel systems, biopolymer-based nanocarriers, micro/nano hybrid systems, and stimuli-responsive platforms, further expand the versatility of nanoencapsulation. Nanoemulsion–hydrogel systems synergistically enhance the solubility, stability, and controlled release of lipophilic bioactive compounds such as curcumin (163), whereas biopolymer-based systems using chitosan, alginate, or gelatin provide safe, biodegradable delivery of compounds such as vitamins and probiotics [199]. Micro/nano hybrid systems integrate nanoparticles into microparticles to enhance protection and prolong release, improving stability and bioavailability [200]. Stimuli-responsive combinatorial systems, including pH-sensitive liposome–polymer hybrids, enable site-specific release in response to environmental triggers, increasing bioavailability and therapeutic effectiveness [201]. Overall, nanoencapsulation provides structural protection, enhanced solubility, targeted delivery, and improved bioavailability across oral and inhalation routes, thereby representing a robust strategy for advancing the development of nutraceuticals, functional foods, and pharmaceuticals.

### 4.3. Personalized Resolution Therapies

Personalized resolution therapies represent a paradigm shift in the management of inflammation from nonspecific suppression to precision-guided resolution. Instead of merely inhibiting inflammatory pathways, these approaches actively harness individual molecular signatures to restore immune homeostasis. By integrating transcriptomic, proteomic, and genomic biomarkers, clinicians can now predict therapeutic response, monitor subclinical disease, and dynamically adapt treatment strategies. This model transforms care in complex immune-mediated diseases such as IBD and cardiovascular disease (CVD), improving remission rates and minimizing adverse events [202,203,204].

#### 4.3.1. Biomarker-Guided Approaches in Inflammatory Bowel Disease

IBD, including Crohn’s disease and ulcerative colitis, is driven by mucosal immune dysregulation. Although biologics target TNF-α, IL-12/23, and integrins, up to 40% of patients still experience non-response or secondary loss [205]. Biomarker-guided personalization can address this therapeutic variability. Biomarkers, surrogate markers for disease activity, include C-reactive protein (CRP) and fecal calprotectin (FC) and have consequently become increasingly adopted non-invasive measurements of IBD activity as an intermediate stage in the treat-to-target method [206].

##### Transcriptomic and Proteomic Markers

Assessment of gene activity in both tissue and blood samples enables the prediction of therapeutic efficacy for specific pharmacologic agents. Elevated *TNF*, *IL23A*, and *CXCL10* expression correlate with anti-TNF responsiveness [207]. Distinct gene modules identified by single-cell RNA sequencing (scRNA-seq) delineate immune subtypes predictive of the anti-IL-23 response. Proteomic markers provide real-time tracking of inflammation: fecal calprotectin and serum CRP reflect neutrophil infiltration and systemic inflammation [208]. Tissue cytokines, including IL-6, IL-8, and IL-33, serve as indicators of tissue healing. Elevated baseline calprotectin levels are associated with poor response to anti-integrin therapy, supporting the rationale for early modification of treatment regimens [209]. Together, these molecular profiles stratify patients into responders and non-responders, enabling the selection of first-line therapy rather than empirical sequencing. Some of the novel biomarkers identified are Oncostatin M, Leucine-rich alpha 2-glycoprotein, fecal myeloperoxidase, and fecal *microRNAs*. Glycome profiling has been used to determine the correct treatment for IBD and CD [210].

##### Minimal Residual Disease Monitoring

Minimal Residual Disease (MRD) refers to the persistence of a limited number of cancer cells, sometimes in the form of DNA fragments or proteins, that remain in the patient’s body during or after treatment despite clinical remission. These remaining cells are undetected by traditional medical imaging, routine screening procedures, or standard histological investigation. The idea of MRD was initially introduced in the context of acute lymphoblastic leukemia and acute myeloid leukemia. Over the past decade, with developments in sensitive detection methods, MRD research has expanded its reach beyond hematologic malignancies to solid tumors, such as breast, colorectal, lung, ovarian, and other cancer types, reflecting its increased prognostic value [211]. The capacity to detect MRD without symptoms provides valuable information that typical techniques may miss. This is critical for assessing therapy efficacy, predicting recurrence, and guiding cancer medicine clinical trial objectives [212].

Several studies have revealed that MRD response to first-line therapy has become an independent predictor beyond standard prognostic measures and has a wide range of practical application prospects. All MRD detection approaches exploit traits that are present solely in leukemic blasts to separate them from normal cells. Commonly used techniques include multicolor flow cytometry (MFC) to detect leukemic cells by immunophenotypic aberrancies, real-time quantitative polymerase chain reaction (qPCR) for the detection of recurrent gene fusions (e.g., BCR-ABL1) or rearranged immunoglobulin (IG) and T-cell receptor (TCR) genes. A more recent technology, which uses high-throughput next-generation sequencing (NGS), may give a more sensitive approach to detect IG and TCR rearrangements in ALL blasts [213].

#### 4.3.2. Biomarker-Guided Approaches in Cardiovascular Disease

By providing objective, quantitative indicators of illness presence and progression, biomarkers have improved cardiovascular disease identification, risk classification, and management. High sensitivity and specificity, especially in acute circumstances, are their advantages. For example, cardiac troponins (cTnI, cTnT) are the gold standard for detecting myocardial infarction, while natriuretic peptides (BNP, NT-proBNP) are used to measure heart failure severity. High-sensitivity C-reactive protein (hs-CRP) is an early indicator of vascular inflammation and is linked to atherosclerosis, while microRNAs and circulating endothelial cells may detect cardiovascular pathology before structural changes [214]. Clinicians use biomarkers such as galectin-3 and soluble ST2 (sST2) to personalize treatment for cardiac remodeling and heart failure progression. Lipoprotein(a) [Lp(a)] is an independent predictor of atherosclerotic cardiovascular disease, enabling more personalized risk assessment, especially in people with a family history of premature coronary artery disease. Most biomarkers can be assessed with standard blood testing, minimizing the need for invasive procedures [215,216,217,218].

##### Metabolomic Approaches in Cardiovascular Disease

Metabolomics examines lipids, carbohydrates, organic acids, amino acids, and other small-molecule analytes that are substrates and byproducts in many metabolic pathways to study medical disorders, including cardiovascular disease [219]. Acylcarnitines, BCAAs [220], glutamate, and ceramides are recurrent metabolites linked to many CVDs, such as myocardial infarction, congestive heart failure, CAD, stroke, transient ischemic attack, peripheral artery disease, and unstable angina [214]. Citrulline levels alone were a substantial and independent predictor of serious adverse cardiac events [221]. Atrial fibrillation is hypermetabolic, with upregulation of glycolytic enzyme transcripts, suggesting glucose consumption [222]. Elevated levels of beta-hydroxybutyrate, ketogenic amino acids (tyrosine and leucine), and glycine were observed in atrial tissue of patients with a history of persistent atrial fibrillation as compared with controls with no prior history of atrial fibrillation. This revealed the importance of ketone bodies as an energy source to perpetuate the arrhythmia [219]. Metabolomics may help identify and diagnose people before significant cardiac events.

### 4.4. Genetic Predisposition and Risk Stratification

Large-scale genetic studies demonstrate that specific variants, such as those in IL6, CRP, and NLRP3, modulate cytokine responses and influence both inflammation and cardiovascular disease risk. *PCSK9* and *APOE* variants affect lipid metabolism and plaque vulnerability [223]. Polygenic risk scores (PRS) combine these loci. They forecast risk and help tailor interventions [224]. When used with inflammatory biomarkers, PRS guides prevention, therapy intensity, and safety monitoring [225].

## 5. Conclusion, Limitations, and Future Perspectives

The growing understanding of inflammation as a dynamic and self-limiting process rather than a merely destructive response has led to a paradigm shift from suppression to active resolution in therapeutic strategies. Conventional anti-inflammatory drugs often provide transient relief by blocking pro-inflammatory mediators such as TNF-α and IL-1β but fail to restore tissue homeostasis, leading to chronic or relapsing conditions. Bioactive compounds derived from plants, marine organisms, and gut microbiota have emerged as promising candidates due to their multitarget mechanisms that modulate oxidative stress, cytokine signaling, and immune cell polarization. Plant-based polyphenols, lignans, and glycyrrhizin act on pathways like NF-κB and MAPK, while marine-derived agents and short-chain fatty acids produced by gut microbes strengthen barrier integrity and regulate macrophage activity. In parallel, the development of pro-resolving mediators such as lipoxins, resolvins, and protectins represents a breakthrough, as they mimic endogenous molecules that actively terminate inflammation and promote tissue repair. Non-pharmacological interventions, including dietary modifications and physical modalities such as cryotherapy or light stimulation, further complement these pharmacological strategies by modulating systemic inflammatory tone. Together, these approaches represent an integrated, resolution-oriented framework that targets both the molecular triggers and the failure of natural resolution mechanisms.

Despite these advances, significant limitations impede the translation of resolution-based strategies into clinical practice. Many bioactive compounds exhibit poor solubility, instability, and limited bioavailability, which compromise their systemic efficacy. Moreover, discrepancies between preclinical and clinical outcomes persist due to species differences, dosage inconsistencies, and inadequate modeling of human inflammatory diseases. The heterogeneity of chronic inflammation, driven by individual genetic, metabolic, and microbial variability, further complicates the predictability of therapy. In addition, the mechanistic understanding of how bioactive compounds interact with pro-resolving pathways, immune subsets, and the microbiome remains incomplete. The absence of standardized extraction, formulation, and characterization techniques also hampers reproducibility and regulatory approval. Addressing these challenges requires a multidisciplinary approach integrating molecular biology, systems pharmacology, and bioengineering to refine compound delivery, standardization, and efficacy testing.

Looking ahead, future research should prioritize enhancing bioavailability through nanocarrier systems, liposomal encapsulation, and combinatorial delivery systems that enable targeted and sustained release. Personalized resolution therapies guided by biomarkers and immune profiling could allow tailored interventions for complex disorders such as inflammatory bowel disease, atherosclerosis, and neurodegeneration. Integrating multi-omics platforms, including metabolomics, transcriptomics, and microbiome analysis, will deepen understanding of the interplay between diet, immune regulation, and inflammation resolution, ultimately improving treatment precision. Advances in biomaterial science, such as cytokine-coated scaffolds or macrophage-polarizing implants, may redefine tissue regeneration by coupling immunomodulation with structural healing. Additionally, lifestyle-centered interventions that synergize dietary fiber intake, polyphenol-rich foods, and controlled physical therapies hold promise. Ultimately, the transition from anti-inflammatory suppression to pro-resolving restoration represents a transformative shift in the management of chronic inflammation, emphasizing not only the cessation of injury but also the reestablishment of immune balance and tissue integrity for long-term health resilience.

## Figures and Tables

**Table 1:** In vitro studies of bioactive compounds.

Natural Compounds/Typical Experimental Dose Range (In Vivo)	Models/Methods	Findings	Reference
**Polyphenols**			
Resveratrol/ 10–100 mg/kg	RAW 264.7 macrophages (LPS-induced)	Inhibited NF-κB nuclear translocation, MAPK phosphorylation, and COX-2/iNOS expression Activated Nrf2/HO-1 antioxidant axis Reduced levels of NO, PGE2, TNF-α, IL-6, and IL-1β Reduced oxidative stress	[32,33]
Lignans/ 20–100 mg/kg	RAW 264.7 macrophages (LPS-induced)	Suppression of IκB-α Inhibited NF-κB Downregulation of COX-2 expression Suppressed NO, PGE2, TNF-α, and IL-6 release in a dose-dependent manner	[34]
**Terpenoids**			
Lycopene/ 5–50 mg/kg	IPEC-J2 intestinal cells and Kupffer cells	Inhibited NLRP3 inflammasome assembly Suppressed caspase-1 Targeting and modulation of the ERK signaling pathway Reduced secretion of IL-1β Reduced secretion of IL-18 (pyroptosis markers) Decreased activity of TNF-α and IL-6	[35,36]
Glycyrrhizin/ 50–150 mg/kg	RAW 264.7 macrophages and HMEC-1 cells	Inhibition of HMGB1 (alarmin) release and receptor binding Prevention of downstream NF-κB signaling activation Prevention of MAPK signaling pathway activation Reduced release of pro-inflammatory mediators TNF-α, IL-1β, and IL-6 Decreased nitric oxide (NO) production Protection against inflammation-induced cellular apoptosis	[37,38]
**Indoles**			
Indole-3-Carbinol/ 20–100 mg/kg	Caco-2 cells and chicken macrophages	Activation of the aryl hydrocarbon receptor (AhR) to regulate mucosal immune responses Inhibition of bacterial adhesion-induced NF-κB activation Reduced levels of pro-inflammatory cytokines IL-1β and IL-8 Increased expression of tight junction proteins Restoration and strengthening of epithelial barrier integrity	[39,40]

**Table 2:** In vivo studies of bioactive compounds.

Natural Compounds	Models/Methods	Findings/Results	Reference
**Polyphenols**			
Resveratrol	1. Diabetic neuropathy model: steptozotocin (STZ)-induced diabetic mice	Increased Nrf2 expression in peripheral nerves Inhibited the NF-κB signaling pathway, leading to reduced apoptosis and improved pain sensitivity	[41]
	2. Acute pharyngitis model: rabbit model induced by specific inflammatory triggers	Suppression of TLR4 expression MyD88 suppression Reduced serum TNF-α Reduced serum IL-6 levels Inhibited NLRP3 inflammasome activation	[42]
Lignans	1. Mammary tumor model: C57BL/6 mice injected with E0771 tumor cells	Reduction in tumor growth following supplementation Decreased macrophage infiltration into tumor tissue Suppression of NF-κB signaling, indicated by reduced phosphorylated p65 (p-p65) Downregulation of Ccl2 expression	[43]
	2. Peritoneal inflammation: asbestosis-induced peritoneal inflammation in mice	Reduced WBC accumulation in peritoneal fluid Increased enterolactone levels in plasma	[44]
Lycopene	1. Hypercholesterolemia: high-cholesterol diet (HCD) fed rats	Correction of renal dysfunction markers Correction of cardiac dysfunction markers Marked upregulation of Nrf2 Increased expression of HO-1 Increased expression of PON-1 Reduced PPAR-γ expression	[45]
	2. Colitis model: TNBS-induced colitis rats	Reduced colonic thickness Decreased myeloperoxidase activity Attenuation of diffuse hemorrhagic necrosis in colonic mucosa Reduced mucosal fibrosis	[46]
Glycyrrhizin	1. Cerebral ischemia/reperfusion: focal cerebral injury in rats	Directly inhibits HMGB1 cytokine activity and release Reduction in infarct volume Suppression of TNF-α expression Inhibition of iNOS expression Decreased IL-1β levels in p38/JNK pathways Modulation of p38 and JNK signaling pathways	[47]
	2. Acute liver injury: LPS-induced acute liver injury in mice	Alleviation of inflammatory marker accumulation, including iNOS, COX-2 Regulation of the PI3K/mTOR signaling pathway Reduction in apoptosis	[38]
Indoles	1. Colitis model: TNBS-induced ulcerative colitis in rats	Mitigation of the severity of colonic lesions Facilitation of mucosal regeneration Activation of tissue repair pathways Reduction in inflammation-associated tissue damage	[39]
	2. Genetic colitis model: AhR-deficient mice exposed to DSS	Restoration of gut microbiota balance with an increasing number of butyrate-producing bacteria Induction of IL-22 production Enhancement of regulatory T-cell (Tregs) populations Maintenance and strengthening of intestinal barrier integrity	[39]

**Table 3:** Analysis of pharmacokinetics challenges and current clinical standing of major bioactives.

Bioactive Compound	Main PK/Translational Barriers	Approach to Enhance Clinical Impact	Relevance in Human/Clinical Studies	Reference
Curcumin	Extremely poor aqueous solubility; rapid elimination through the glucuronidation process.	Nanomicelle and phospholipid complexes to avoid first-pass metabolism.	Currently undergoing multiple phase II/III studies for metabolic disorders; requires high dosage or nanodelivery system.	[123]
Resveratrol	Elevated absorption rate but extremely poor bioavailability (~<1%) owing to rapid metabolic breakdown.	Micronization technique or lipid–polymer hybrid nanoparticles for delivery.	Demonstrated promise in enhancing insulin sensitivity; efficacy is heavily dose-dependent.	[124]
Quercetin	Poor gastric stability; highly dependent on microbial activation for utilization	Embedding into a pH-sensitive hydrogel or pectin-based carrier system.	Demonstrated potential in improving insulin sensitivity; bio-efficacy is highly dose-dependent.	[125]
Rutin	Heavy molecular weight; poorly absorbable without microbial activation through α-rhamnosidase enzyme.	Prebiotic delivery system to influence “microbial gatekeeper” function.	Great potential in vascular health; clinical efficacy is highly dependent on the individual microbiome.	[126]
Lycopene	Hypersensitive to lipid-based foods; high lipophilicity	Self-emulsification drug delivery system (SEDDS); nanoemulsion technology.	Very strong epidemiological evidence for prostate and cardiovascular health; needs a stable formulation.	[127]
Epigallocatechin gallate (EGCG)	Poor oxidation resistance; poor absorption due to poor intestinal permeability; poor stability at physiological pH.	Nanocarrier delivery using protein (e.g., casein/zein) protection.	Extensive human studies; requires protection to retain “pro-resolving” properties in blood circulation.	[128]

## Data Availability

No new data were created or analyzed in this study.
